# Calving outcomes after ipsilateral versus contralateral supplementary embryo transfer in repeat-breeder Holstein cows

**DOI:** 10.1590/1984-3143-AR2026-0007

**Published:** 2026-08-28

**Authors:** Chihiro Kanno, Takahiro Gyoda

**Affiliations:** 1 Laboratory of Clinical Veterinary Medicine for Large Animals, School of Veterinary Medicine, Kitasato University, Towada, Japan.; 2 Tokachi AI Association, Obihiro, Japan.

**Keywords:** artificial insemination, calving rate, repeat breeder, supplementary embryo transfer, twin calving

## Abstract

Supplementary embryo transfer (ET) following artificial insemination (AI) is used in repeat-breeder cows; however, this dual-embryo approach inherently increases the risk of twin outcomes. This study evaluated whether transferring an embryo to the uterine horn contralateral to the corpus luteum (CL) could be associated with different calving outcomes from ipsilateral transfer. Holstein cows (n = 187; 79 nulliparous and 108 multiparous) received ipsilateral or contralateral ET 7–8 days after AI using frozen–thawed, in vitro-produced embryos. Repeat-breeder cows were clinically defined as cows failing to conceive after ≥3 artificial inseminations without evident reproductive abnormalities on routine examination. Data were analyzed using a generalized linear mixed model accounting for parity and technician. The calving rate was 34.8% (32/92) in the contralateral group and 46.3% (44/95) in the ipsilateral group, with no significant difference between groups (aOR = 0.62, 95% CI 0.33–1.15; P = 0.137). Twin calving incidence was numerically lower after contralateral ET (4.8%, 1/21) compared with ipsilateral ET (21.2%, 7/33; P = 0.131). Because this study was based on calving records and involved a limited number of animals, the findings should be interpreted cautiously. Contralateral supplementary ET may represent a practical option for further investigation.

## Introduction

Repeat-breeder cows present a major challenge in dairy and beef reproduction, with low conception rates despite multiple artificial inseminations (AI) (Carbonari et al., 2024). In Japan, a unique clinical approach known as "supplementary embryo transfer (ET)"—transferring an embryo 7 days after AI—is widely applied to reduce early embryonic loss and improve conception, particularly in cows with a history of repeat breeding (Dochi et al., 2008; Yaginuma et al., 2019). While this dual-embryo approach may improve reproductive outcomes, it may also increase twin outcomes when both the AI-derived and transferred embryos survive.

The use of beef semen in dairy herds has increased worldwide to improve calf market value, a system known as beef-on-dairy (Berry, 2021). However, pregnancy rates following AI with beef semen can be suboptimal in repeat-breeders, highlighting the need for practical strategies to balance reproductive performance with the risks of multifetal gestation. In conventional embryo transfer programs, embryos are generally transferred to the uterine horn ipsilateral to the corpus luteum (CL). Previous studies have shown that embryos can survive after transfer to either the ipsilateral or contralateral uterine horn (Tervit et al., 1977; Christie et al., 1979; Del Campo et al., 1983). In the context of supplementary ET, transfer laterality may be relevant because an AI-derived embryo and a transferred embryo may coexist within the same cycle. However, the biological basis for any effect of transfer side on subsequent outcomes is likely complex. Studies of accessory corpora lutea during pregnancy indicate that conceptus-mediated maintenance of luteal function has a substantial local component, because contralateral accessory corpora lutea are more likely to regress than ipsilateral accessory corpora lutea (Baez et al., 2017; Monteiro et al., 2021a, b). In addition, the uterine environment and endometrial response to a conceptus differ between uterine horns ipsilateral and contralateral to the CL (Sánchez et al., 2019; Bagés-Arnal et al., 2020). Therefore, transfer laterality could plausibly influence the maintenance of one or both embryos in supplementary ET, although the underlying mechanisms remain uncertain and available evidence is still limited. Twin births are associated with dystocia, stillbirth, and significant economic loss (Moore and Hasler, 2017). Despite its potential, few studies have systematically compared ipsilateral and contralateral ET specifically within a supplementary ET protocol under routine field conditions.

The aim of this retrospective field study was to compare calving-based outcomes after ipsilateral versus contralateral supplementary ET in repeat-breeder Holstein cows under routine commercial conditions.

## Methods

### Supplementary ET after AI and the data collection

From February 2018 to May 2019, 187 Holstein cows were enrolled from commercial dairy herds associated with the Tokachi AI Association Youth Division. In this study, repeat-breeder cows were clinically defined as cows that failed to conceive after three or more artificial inseminations and had no evident reproductive abnormalities on routine veterinary examination. Cows were classified as nulliparous (n = 79) or multiparous (n = 108), and the mean number of previous AI attempts in the supplementary ET cohort was 5.3 ± 1.6. Embryos were transferred 7–8 days after AI to the uterine horn ipsilateral or contralateral to the CL. All transfers involved frozen–thawed, in vitro-produced embryos from the same parental origin as the preceding AI. AI and ET were conducted by eight technicians with over 5 years of experience. Pregnancy diagnosis during gestation was not available in this retrospective dataset; therefore, outcomes were assessed using calving records. Calving rate was defined as the proportion of cows that subsequently calved after the indexed AI and supplementary ET event, and twin calving incidence, dystocia, and stillbirth were recorded from the same records. For descriptive context, aggregate calving rate and twin rate data were also obtained for cows that underwent three or more artificial inseminations without supplementary ET in the same region and period (mean AI attempts, 4.9 ± 1.3), but these were used only as descriptive reference data and were not included in the primary statistical comparisons. All reproductive procedures were performed as part of routine commercial practice. Ethical approval was not required, as this study was a retrospective analysis of routine clinical records without experimental intervention.

### Statistical analysis

Calving rate was analyzed using a generalized linear mixed model (GLMM) with ET side and parity as fixed effects and technician as a random effect. The interaction between ET side and parity was initially included but removed from the final model as it was not statistically significant (P = 0.45). Twin calving incidence, dystocia rate, and stillbirth rate were compared between groups using Fisher’s exact test. Adjusted odds ratios (aORs) with 95% confidence intervals (CIs) were calculated to quantify the association between ET side and each outcome. A P-value < 0.05 was considered statistically significant. Statistical analyses were performed using JMP version 17.0.0 (SAS Institute Inc., Cary, NC, USA).

## Results

The random effect of the technician in the GLMM showed minimal variance, indicating that the operator’s influence on calving rate was negligible. As shown in Table 1, the GLMM analysis revealed no significant difference in calving rate between ipsilateral and contralateral ET (aOR = 0.62, 95% CI 0.33–1.15; P = 0.137), regardless of parity (P = 0.356). Calving rate was numerically lower in the contralateral group (34.8%, 32/92) than in the ipsilateral group (46.3%, 44/95).

**Table 1:** Factors associated with calving rates in Holstein repeat-breeder cows (n = 187) analyzed by a generalized linear mixed model.

Variable	Category	n	Calving Rate (%)	Adjusted Odds Ratio (95% CI)	P value
ET Side	Ipsilateral	95	46.3 (44/95)	1.00 (Reference)	-
	Contralateral	92	34.8 (32/92)	0.62 (0.33 – 1.15)	0.137
Parity	Nulliparous	79	44.3 (35/79)	1.00 (Reference)	-
	Multiparous	108	38.0 (41/108)	0.75 (0.39 – 1.43)	0.356

The model included the random effect of technicians. No significant interaction was observed between ET side and parity (P = 0.45).

Twin calving incidence was numerically lower after contralateral ET than after ipsilateral ET (4.8%, 1/21 vs. 21.2%, 7/33; OR = 0.19, 95% CI 0.02–1.63; P = 0.131), whereas dystocia and stillbirth did not differ significantly between groups (Table 2). As descriptive reference data, AI-only cows showed calving rates of 36.4% (71/195) in nulliparous cows and 31.7% (65/205) in multiparous cows, with twin rates of 4.2% (3/71) and 6.2% (4/65), respectively.

**Table 2:** Twin calving incidence and calving outcomes following ipsilateral versus contralateral supplementary embryo transfer in repeat-breeder Holstein cows.

Outcome	ET side	n	No. of events (%)	Odds Ratio (95% CI)	P value
Twin calving	Ipsilateral	33	7 (21.2%)	1.00 (Reference)	0.131
	Contralateral	21	1 (4.8%)	0.19 (0.02-1.63)	
Dystocia	Ipsilateral	33	2 (6.1%)	1.00 (Reference)	0.638
	Contralateral	21	2 (9.5%)	1.63 (0.21-12.57)	
Stillbirth	Ipsilateral	33	5 (15.2%)	1.00 (Reference)	1.00
	Contralateral	21	3 (14.3%)	0.93 (0.20-4.39)	

For all outcomes, n represents cows with available calving records.

Outcomes were compared between groups using Fisher’s exact test. Odds ratios (ORs) and 95% confidence intervals (CIs) are shown relative to the ipsilateral group (Reference = 1.00). 

## Discussion

In this retrospective field study, contralateral supplementary ET was associated with a numerically lower twin calving incidence than ipsilateral ET in repeat-breeder Holstein cows, although calving rate was also numerically lower and neither difference reached statistical significance. Therefore, the findings should be interpreted cautiously.

Previous studies have shown that pregnancy can be established after embryo transfer to either the uterine horn ipsilateral or contralateral to the CL (Tervit et al., 1977; Christie et al., 1979; Del Campo et al., 1983). In the present study, calving rate after contralateral ET was not significantly different from that after ipsilateral ET, although the point estimate suggested a numerically lower rate in the contralateral group. Because this was a field-based observational study, these results should not be interpreted as evidence that transfer laterality has no biological effect on pregnancy establishment.

One possible explanation is that transfer laterality may influence the likelihood that both the AI-derived and transferred embryos are maintained to term. However, this remains speculative. Studies in cattle suggest that conceptus-mediated support of luteal function has a substantial local component, and endometrial responses differ between uterine horns ipsilateral and contralateral to the CL (Baez et al., 2017; Monteiro et al., 2021a, b; Sánchez et al., 2019; Bagés-Arnal et al., 2020). Thus, any effect of transfer side in supplementary ET is likely biologically complex rather than uniform.

This study has several limitations. It was based on calving records and did not include pregnancy diagnosis during gestation, so reduced twin delivery cannot be distinguished from subsequent embryonic or fetal loss. The sample size, particularly for twin calving outcomes, was limited. In addition, no individual-level AI-only control group was included in the primary analysis, and the AI-only data were available only as aggregate descriptive reference data.

Nevertheless, because contralateral ET requires only a change in transfer side, it may represent a practical option for further evaluation. Larger prospective studies with serial ultrasonographic examination are needed.

## Conclusion

In conclusion, contralateral supplementary embryo transfer was associated with a numerically lower twin calving incidence than ipsilateral transfer in repeat-breeder Holstein cows, although calving rate was also numerically lower and neither difference reached statistical significance. Because this retrospective study was based on calving records and a limited number of animals, the findings should be interpreted cautiously. Larger prospective studies are needed.

## Data Availability

Research data is only available upon request.
